# Activation of Akt protects cancer cells from growth inhibition induced by PKM2 knockdown

**DOI:** 10.1186/2045-3701-4-20

**Published:** 2014-04-15

**Authors:** Xiaodong Qin, Yuping Du, Xing Chen, Wuyan Li, Jinghong Zhang, Jinbo Yang

**Affiliations:** 1 School of Life Science, Lanzhou University, Lanzhou, Gansu 730000, P. R. China; 2 Department of Engineering Science, Muskingum University, 163 Stormont St, New Concord, OH 43762, USA; 3 School of Biomedical Science and Institutes of Molecule Medicine, Huaqiao University, Xiamen, Fujian 361021, P. R. China; 4 Department of Cancer Biology, Lerner Research Institute, The Cleveland Clinic Foundation, 9500 Euclid Avenue, Cleveland OH 44195, USA

## Abstract

**Background:**

PKM2 is an attractive target for cancer therapy, however, for many cancer cells, PKM2 knockdown only leads to a modest impairment of survival and proliferation. It is not known whether PKM2 knockdown rewires cell signaling pathways in these “PKM2 knockdown resistant” cells, and whether the rewired pathways are needed for their survival.

**Findings:**

In present study, we investigated the effects of PKM2 knockdown on cellular signaling pathways in “PKM2 knockdown resistant” cancer cells. We found that knockdown of PKM2 leads to activation of Akt. Furthermore, we revealed that activation of Akt in PKM2 knockdown cells is a result of glycolysis disruption. Inhibiton of PI3K-Akt signaling pathway leads to significant growth inhibition and apoptosis in PKM2 knockdown cells.

**Conclusions:**

Overall, our results indicate that activation of Akt is necessary for the survival of PKM2 knockdown cells. Combing PKM2 knockdown with PI3K or Akt inhibitors may lead to a better chance to kill tumors. Our research may provide an unexpected opportunity for the development and implementation of drugs targeting cell metabolism and aberrant Akt signaling.

## Findings

### H1299 cells are resistant to PKM2 knockdown induced growth inhibition

**Figure 1:**
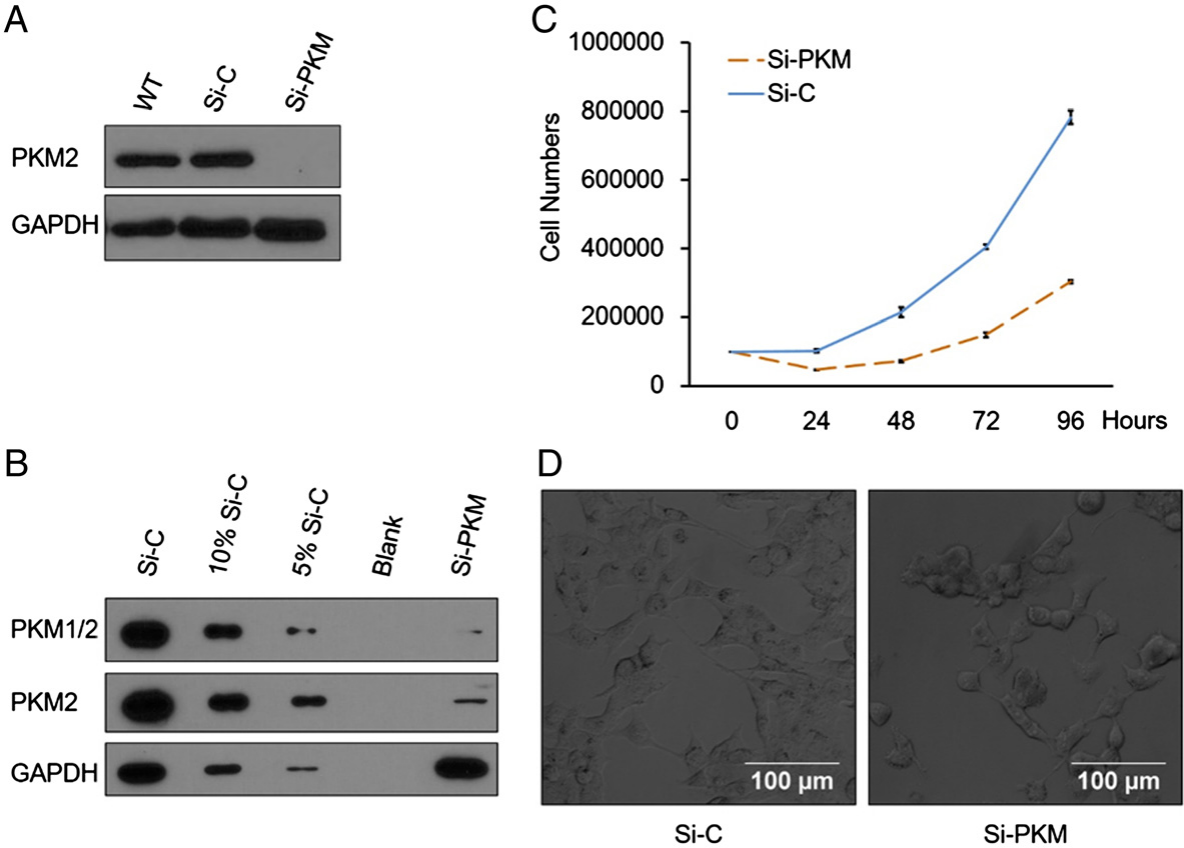
**H1299 cells are resistant to PKM2 knockdown induced growth inhibition. (A)** Knockdown of PKM2 in Si-PKM cells was confirmed by Western blot. GAPDH was used as equal loading control. **(B)** PKM2 knockdown efficiency in Si-PKM cells was higher than 95%. Si-C cell lysate was diluted to 10% and 5%. PKM2 in different dilutions were analyzed by immunoblotting in compare with Si-PKM cell lysate. **(C)** Maximal proliferation rates of Si-C and Si-PKM cells are similar. Si-C and Si-PKM cells were seeded into 6-well plates and cell counts were obtained every 24 h for 4 days. Data are shown as means ± SEM. n = 3 **(D)** Imaging of Si-C and Si-PKM cells with phase-contrast microscope.

To knockdown PKM2, we introduced a PKM2 specific shRNA into a variety of human cancer cell types. Empty vector (pLKO.1) served as control. After stable cells were obtained, we checked whether PKM2 is silenced in our stable cells by western blot. Taking H1299 cells as an example, as shown in Figure 1A, PKM2 in H1299 Si-PKM cells was greatly reduced. Protein dilution experiment showed the knockdown efficiency in Si-PKM cells is higher than 95% at protein level (Figure 1B); further quantification with Image J showed the knockdown efficiency is about 98%. Even with such a high knockdown efficiency, we did not observe significant difference in the maximal proliferation rate between Si-C cells and Si-PKM cells (Figure 1C). Morphologically, Si-C cells were different from Si-PKM. While Si-C cells displayed an epithelioid appearance growing adherent to the plastic surface. In marked contrast, Si-PKM cells assumed a spherical shape (Figure 1D).

### PKM2 knockdown induces activation of Akt signaling pathway

**Figure 2:**
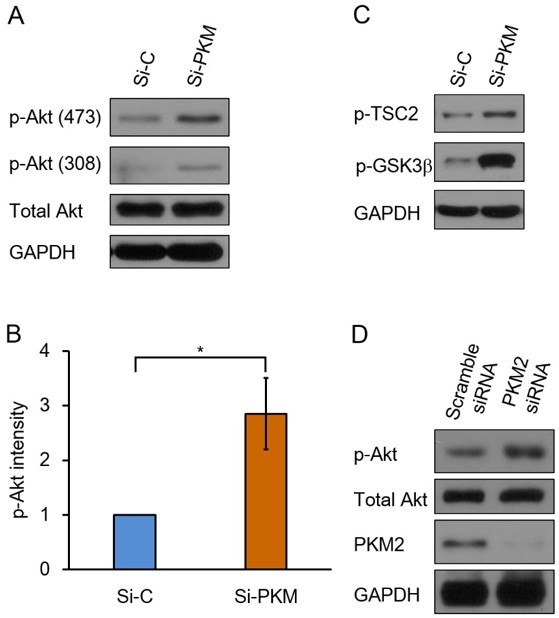
**PKM2 knockdown induces activation of Akt. (A)** Si-C and Si-PKM cell lysates were analyzed by immunoblotting for phosphorylation of Akt at Thr308 and Ser473. GAPDH was used to verify equal gel loading. **(B)** The p-Akt levels were normalized to the loading control and presented as relative conversion to values in Si-C cells. Data are shown as means ± SEM. n = 3. Statistical analyses were carried out using Student’s t-test. Significance: *p < 0.05; **p < 0.001. **(C)** Si-C and Si-PKM cell lysates were analyzed by immunoblotting with antibodies against phospho-TSC2 and phospho-GSK3β. GAPDH was used as an equal loading control. **(D)** H1299 cells were transfected with 20 nM of scramble siRNA and PKM2-specific siRNA, respectively. 48 hours after transfection, cells were harvested and analyzed by immunoblotting with the following antibodies: p-Akt, total Akt and PKM2. GAPDH was used as an equal loading control. Similar results were obtained in three independent experiments. Representative data are shown.

To investigate possible changed signaling pathways in Si-PKM cells,we tested the activation status of PI3K-Akt signaling pathway, one of the most frequently deregulated signaling pathways in cancers [1,2]. Akt activation involves the phosphorylation of two residues: threonine 308 (Thr308) and serine 473 (Ser473). As shown in Figure 2A, phosphorylated Akt (p-Akt) was significantly increased in Si-PKM cells, while total Akt was not changed. We quantified p-Akt intensity with Image J, p-Akt level was about 3 folds higher in Si-PKM cells (Figure 2B). Activated Akt has been shown previously to phosphorylate GSK3β at Ser9 and TSC2 at Thr1462. Indeed, in Si-PKM cells, phosphorylation of GSK3β and TSC2 were also increased (Figure 2C). Then, we tried anther strategy to knockdown PKM2 in H1299 cells. Transfection of H1299 cells with a PKM2 specific siRNA also led to a significant decrease of PKM2 and an increase of p-Akt (Figure 2D). We also tested generalization of PKM2 knockdown induced Akt phosphorylation, in A549, HCT116 and SW480 cells, PKM2 knockdown all led to increased Akt phosphorylation (Additional file 1: Figure S1). In “PKM2 knockdown sensitive” cells, such as MB-MDA-231 and HepG2 cells, PKM2 knockdown efficiencies are poor, and in these cells we did not observed a significant increase in p-Akt (Additional file 2: Figure S2).

### Activation of Akt signaling pathway in PKM2 knockdown cells is a result of glycolysis disruption

**Figure 3:**
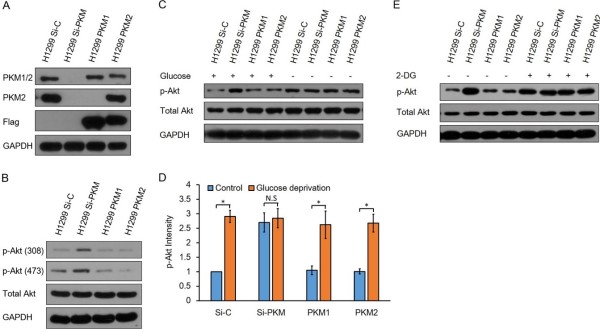
**Activation of Akt is a result of glycolysis disruption. (A)** Using a lentiviral protein expression vector, we reconstituted the expression of PKM1 or PKM2 in Si-PKM cells. Cell lysates were analyzed by immunoblotting with the following antibodies: PKM1/2, PKM2 and Flag. GAPDH was used as an equal loading control. **(B)** Re-expression of either PKM2 or PKM1 normalized p-Akt to similar level as that of Si-C cells. **(C)** Si-C cells, Si-PKM cells, PKM1 cells and PKM2 cells were kept in high glucose DMEM or glucose-null DMEM for 2 hours, cell lysates were analyzed by immunoblotting with antibodies against p-Akt, total Akt and GAPDH. Similar results were obtained in three independent experiments. Representative data are shown. **(D)** The p-Akt levels were normalized to the loading control and presented as relative conversion to values in Si-C cells with high glucose DMEM. Data are shown as means ± SEM. n = 3. Statistical analyses were carried out using Student’s t-test. Significance: *p < 0.05; **p < 0.001. **(E)** Si-C cells, Si-PKM cells, PKM1 cells and PKM2 cells were treated with 25 mmol/L 2-DG or left untreated for 2 hours, cell lysates were analyzed by immunoblotting with antibodies against p-Akt, total Akt and GAPDH. Similar results were obtained in three independent experiments. Representative data are shown.

Then, we reconstituted PKM2 and PKM1 in Si-PKM cells, respectively; we named PKM2 reconstituted Si-PKM cells PKM2 and PKM1 reconstituted Si-PKM cells PKM1 (Figure 3A). When expression of PKM2 was restored in Si-PKM cells, p-Akt decreased to a similar level as that of Si-C cells (Figure 3B). These data clearly show that up-regulation of p-Akt is a bona fide result of PKM2 knockdown. We also found that PKM1 expression could normalize p-Akt to a similar level as that of Si-C cells. PKM1 is a constitutive active isoform of pyruvate kinase and has not been found to play roles other than catalyzing PEP to pyruvate [3], so expression of PKM1 could only restore pyruvate kinase activity and glycolysis in the cells. The observation that expression of PKM1 could decrease p-Akt suggests that up-regulation of p-Akt in Si-PKM cells is due to glycolysis disruption. To futher test this hypothesis, we tested the effect of glucose starvation, a strategy that disrupt glycolysis, on p-Akt in Si-C and Si-PKM cells. Consistence with previous studies, glucose starvation for 2 hours led to up-regulation of p-Akt in Si-C cells [4]. However, p-Akt in Si-PKM cells stayed unchanged in response to glucose starvation (Figure 3C and D), indicating that Si-PKM cells are already in a state that mimics glycolysis disruption. We also test the effect of 2-DG, a glycolysis inhibitor, on Akt phosphorylation. Similar to glucose deprivation, 2-DG treatment also led to increased p-Akt in Si-C cells but not in Si-PKM cells (Figure 3E). Combing these evidences, we conclude that activation of Akt in PKM2 knockdown cells is a result of glycolysis disruption.

### Inhibition of PI3K-Akt signaling pathway induces growth inhibition and cell death in Si-PKM cells

**Figure 4:**
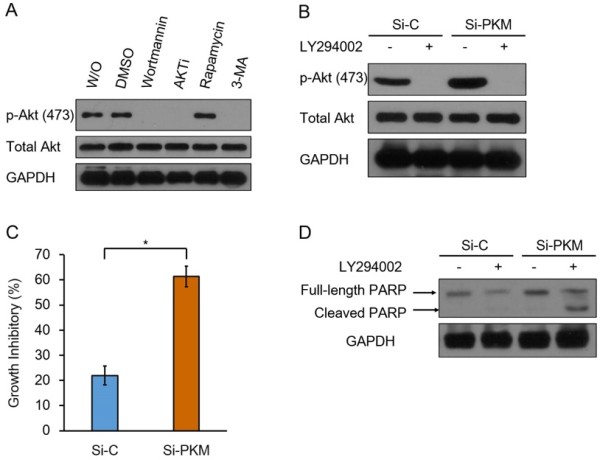
**Inhibition of PI3K-Akt signaling pathway induces growth inhibition and cell death in Si-PKM cells. (A)** Si-PKM cells in 6-well dish were treated with: DMSO, 1 μM Wortmannin, 1 μM AKTi, 100 nM Rapamycin and 5 mM 3-MA respectively for 2 hours. Cell lysates were then analyzed by Western blot with antibodies against p-Akt and total Akt. GAPDH was used as an equal loading control. **(B)** Si-C and Si-PKM cells were treated with 10 μM LY294002 for 24 hours or left untreated, Cell lysates were analyzed by Western blot with antibodies against p-Akt and total Akt, GAPDH was used as an equal loading control. **(C)** Si-C and Si-PKM cells in 96-well plate were treated with 10 μM LY294002 or left untreated, plates were subjected to MTT assay after 24 h. Reactions were carried out in triples. Data are shown as means ± SEM. n = 3. Statistical analyses were carried out using Student’s t-test. Significance: *p < 0.05; **p < 0.001. **(D)** Si-C and Si-PKM cells in 6 well plate were treated with 10 μM LY294002 for 24 hours or left untreated, cell lysates were analyzed by Western blot with antibodies against PARP. GAPDH was used as an equal loading control. Similar results were obtained in three independent experiments. Representative data are shown.

Because Akt activation promotes cell survival, proliferation and inhibits apoptosis, up-regulation of p-Akt may be necessary for the survival of PKM2 knockdown cells. To investigate this possibility, we first evaluated sensitivity of p-Akt to different metabolic and signaling pathway inhibitors in Si-PKM cells. As shown in Figure 4A, both PI3K inhibitor wortamannin and autophagy inhibitor 3-MA could inhibit phosphorylation of Akt in Si-PKM cells, suggesting that phosphorylation of Akt in Si-PKM cells is through the canonical PI3K-Akt signaling pathway and is maintained by autophagy. Then we performed a growth inhibition assay with another PI3K inhibitor LY294002, whose half-life is much longer than Wortamannin. LY294002 was sufficient to block Akt activation in both Si-C and Si-PKM cells (Figure 4B). After 24 hours of treatment, LY294002 led to a 60% growth inhibition in Si-PKM cells; whereas the inhibitory effect in Si-C cells was much less significant. (Figure 4C). Consistent with this, cleaved PARP, a marker of apoptosis, significantly increased in LY294002 treated Si-PKM cells but not in Si-C cells (Figure 4D). Thus, PKM2 knockdown cells rely on PI3K-Akt signaling pathway for survival and proliferation.

## Discussion

Pyruvate kinase, especially PKM2, has been shown to play an important role in the reprogramming and maintenance of altered metabolism in cancer cells [5]. In addition to metabolic reprogramming, PKM2 has also been demonstrated to directly regulate gene expression and subsequent cell cycle progression [6]. At the same time, many oncogenic signaling pathways promote the metabolic reprogramming of cancer cells. It is now well accepted that systems-level cross-talk between metabolism and signaling pathways is required in the maintenance of cancer cell homeostasis [7].

Akt is a positive regulator of PKM2. Akt activation leads to activation of mTOR1 through phosphorylation and inhibition of TSC2; mTOR activation induces HIF1α expression, and HIF1α in turn enhances PKM2 expression through collaboration with c-Myc–hnRNPs splicing regulators [8]. Here we show that loss of PKM2 leads to activation of Akt signaling pathway. Activation of Akt signaling pathway in PKM2 knockdown cells is a result of glycolysis disruption, and is through the canonical PI3K-Akt signaling pathway.

Activation of Akt plays important roles in cancer resistance to different therapeutics [9]. Here we show that resistance of cancer cells to PKM2 deprivation mediated cell death is also mediated by Akt signaling pathway. PI3K inhibitor suppress proliferation of PKM2 knockdown H1299 cells and induces apoptosis. PKM2 knockdown renders cancer cells exquisitely sensitive to Akt inhibition. This indicates PKM2 knockdown cells relies on p-Akt for their survival and proliferation.

In conclusion, we have established a potential role of PI3K-Akt signaling pathway for survival of PKM2 knockdown cancer cells. Combining PKM2 knockdown with Akt or PI3K inhibitor leads to a better chance to kill cancer cells. Thus, cancer signaling pathways should be taken into account when targeting metabolic pathways in treating cancers.

## Competing interests

The author has no competing interest.

## Authors' contributions

XQ, YD and XC conceived and executed the experimental procedures. XQ and WL drafted the manuscript. JY and JZ supervised the project. All authors read and approved the final manuscript.

## Supplementary Material

Additional file 1: Figure S1 PKM2 knockdown led to increased p-Akt in A549, SW480 and HCT116 cells. Cell lysates from Si-C and Si-PKM cells were analyzed by immunoblotting for p-Akt, total Akt and PKM2. GAPDH served as an equal loading control. Click here for file

Additional file 2: Figure S2 PKM2 knockdown didn’t cause p-Akt up-regulation in MB-MDA-231 and HepG2 cells. Cell lysates from Si-C and Si-PKM cells were analyzed by immunoblotting with antibodies against p-Akt, total Akt and PKM2. GAPDH served as an equal loading control. Click here for file
