# Supplementary figures and images for: Activation of Akt protects cancer cells from growth inhibition induced by PKM2 knockdown
Source: Cell Biosci. 2014 Apr 15;4:20. doi: 10.1186/2045-3701-4-20 (PMC4108064; doi:10.1186/2045-3701-4-20)

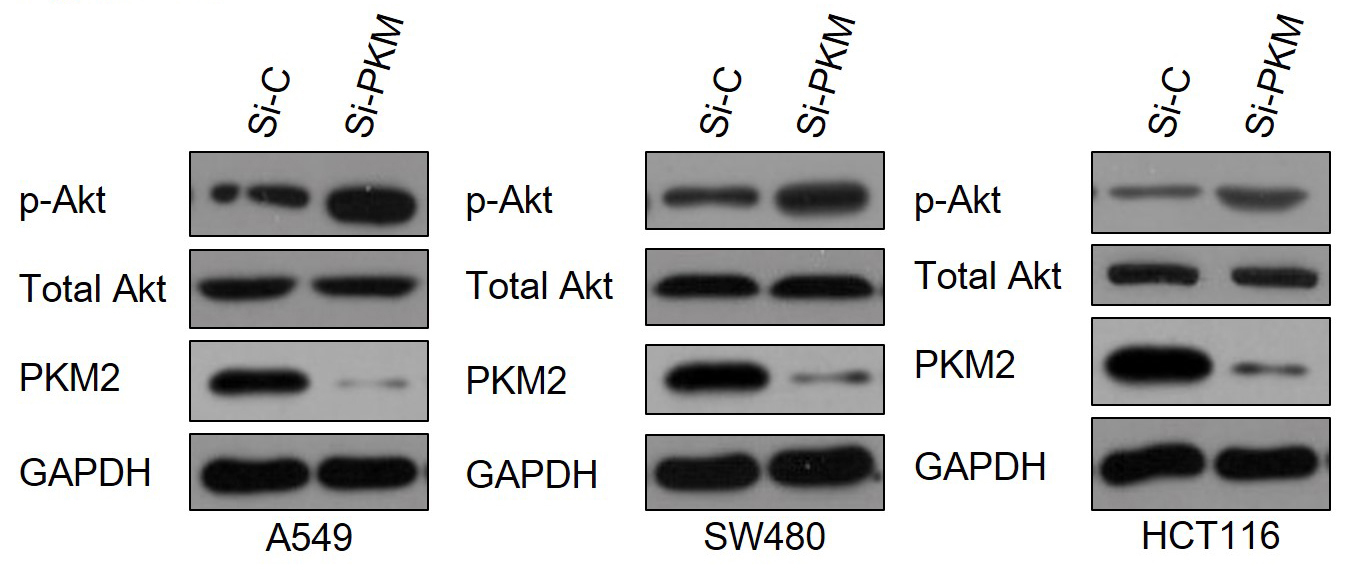

Supplement: Additional file 1: Figure S1 — PKM2 knockdown led to increased p-Akt in A549, SW480 and HCT116 cells. Cell lysates from Si-C and Si-PKM cells were analyzed by immunoblotting for p-Akt, total Akt and PKM2. GAPDH served as an equal loading control. [file 2045-3701-4-20-S1.jpeg]

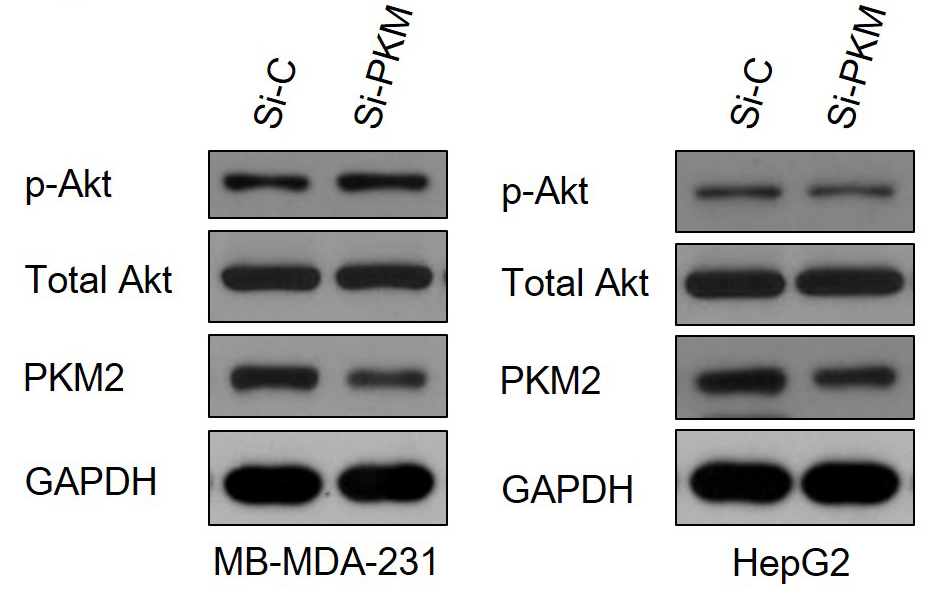

Supplement: Additional file 2: Figure S2 — PKM2 knockdown didn’t cause p-Akt up-regulation in MB-MDA-231 and HepG2 cells. Cell lysates from Si-C and Si-PKM cells were analyzed by immunoblotting with antibodies against p-Akt, total Akt and PKM2. GAPDH served as an equal loading control. [file 2045-3701-4-20-S2.jpeg]
